# A single-arm feasibility phase II study of EMF (erlotinib + methotrexate + 5-fluorouracil) regimen in platinum-refractory recurrent/metastatic head and neck squamous cell carcinoma (R/M HNSCC)

**DOI:** 10.3332/ecancer.2022.1451

**Published:** 2022-09-29

**Authors:** Annie Kanchan Baa, Atul Sharma, Suman Bhaskar, Ahitagni Biswas, Sacchidanand JeeBharti, Alok Thakar, Rajeev Kumar, Raja Pramanik

**Affiliations:** 1 Department of Medical Oncology, Dr B. R. A. Institute Rotary Cancer Hospital, All India Institute of Medical Sciences, New Delhi 110029, India; 2 Department of Radiation Oncology, Dr B. R. A. Institute Rotary Cancer Hospital, All India Institute of Medical Sciences, New Delhi 110029, India; 3 Department of Oncoanesthesia and Palliative Medicine, Dr B. R. A. Institute Rotary Cancer Hospital, All India Institute of Medical Sciences, New Delhi 110 029, India; 4 Department of Head and Neck Surgery, All India Institute of Medical Sciences, New Delhi 110 029, India

**Keywords:** head and neck cancer, recurrent, erlotinib, methotrexate, QOL

## Abstract

**Background:**

Head and neck squamous cell carcinoma (HNSCC) is a huge burden in India with the majority of patients presenting in advanced unresectable stages. Innovative, low-cost but efficacious regimens that can be easily administered in the outpatient setting are the need of the hour. We envisaged assessing whether a readily available triplet therapy of erlotinib + methotrexate + 5-fluorouracil (EMF) is efficacious in terms of extending life and maintaining the quality of life in such patients.

**Patients and methods:**

This was a single-arm, phase II, investigator-initiated interventional study. Thirty-five platinum-resistant/refractory patients of HNSCC were treated with a combination of erlotinib 150 mg, methotrexate 40 mg/m2 (d1, d8) and 5-fluorouracil 500 mg/m2 (d1, d8) every 28 days till progression or unacceptable toxicities. The primary endpoint was overall response rates (ORRs) at 3 months; additional endpoints were disease control rate (DCR) at 3 months, overall survival (OS) and progression-free survival (PFS), safety and patient-reported quality of life.

**Results:**

The ORR and DCR at 3 months were 45.7% and 68.5%, respectively. The PFS was 5 months (95% confidence interval (95% CI): 3.9–6 months) and the OS was 9 months (95% CI: 7.4–10.5 months). The 3- and 6-month PFS rates were 86% ± 6% and 45% ± 9%, respectively, while the OS rates at 3 and 6 months were 91% ± 5% and 68% ± 8%, respectively. Rash, mucositis and fatigue were common adverse events occurring in 23 (65%), 14 (40%) and 9 (25.7%), respectively. The most common grade 3 events seen were rash in 5 (14.2%) and diarrhoea in 2 (5.7%). Clinically significant improvement from baseline was seen in many domains of Quality of Life Core Questionnaire and Quality of Life Head and Neck Module.

**Conclusions:**

The triplet regimen of EMF is a feasible and safe therapeutic option in patients with platinum-resistant/refractory HNSCC. It has demonstrated favourable response rates and improvement in quality of life; however, a randomised phase III study would add more robust value (NCT: CTRI/2020/02/023378).

## Introduction

Developing countries like India are overburdened with increased morbidity and mortality rates that head and neck squamous cell carcinoma (HNSCC) pose [1–3]. Lip and oral cavity cancer is the leading cancer in males (16%) while the fourth most common in females (4.6%) and amounts to 10% mortality field [3]. Unfortunately, a majority (two-thirds) of patients present in advanced stages leading to inoperability [1]. Despite significant advancements in cancer therapeutics and resectability, relapse rates are higher in the rest (one-third) amounting to ≈50% after curative therapy in the first 2 years [1, 4, 5]. In a resource-constrained setting like ours, with a high burden of disease, patients are eventually eligible only for palliative chemotherapy (mostly platinum-based) and radiotherapy on recurrence [6–8]. Targeted therapy (cetuximab) and immunotherapy (pembrolizumab and nivolumab), though designated as standard regimens remain out of reach for the majority with only <1% affording it [9, 10]. Low socioeconomic strata are the most commonly affected population owing to the rampant use of tobacco and smoking [11, 12].

Patients progressing within 6 months of platinum therapy have an aggressive disease causing decreased survival and leaving behind limited choices for further therapy [9, 13]. This led to the ardent need for alternatives in HNSCC. Checkpoint inhibitors have been shown to increase overall survival (OS) (8–10 months) in recurrent settings too, but it comes at a cost of financial toxicity [14]. Prospective randomised studies to prove which regimen would be better are lacking. Oral metronomic therapy has been widely studied and used; with methotrexate being the backbone and the addition of celecoxib augmenting its antiproliferative action [1]. But most of these patients are already on pain medications and NSAIDs (non-specific COX inhibitors); the anti-tumour effect of only celecoxib (selective COX2 inhibitor) is questionable with concomitant use. There is an increased risk of toxicities with concurrent administration [15].

Innovative, low-cost but efficacious regimens that can be easily dispensed without clogging the day care are the need of the hour. Also, recurrent HNSCC patients predominantly present with pain, trismus, chewing and swallowing difficulties posing a significant challenge for oral intake [16, 17]. We designed a 3-drug regimen of intravenous erlotinib, methotrexate and 5-fluorouracil (EMF); wherein all have single-agent activity in HNSCC [11, 18]. The dosing and schedule were extrapolated from the good old Cyclophosphamide/Methotrexate/5-Fluorouracil (CMF) regimen popular in breast cancer [19, 20]. The combination is readily available, cheaper and can be easily administered in OPD settings, with intravenous administration ensuring compliance. We envisaged assessing whether this triplet therapy is superior in terms of extending the life and maintaining the quality of life in such patients. If proven better in efficacy with good tolerability, this EMF regimen could be recommended in low-resource settings all over the world in R/M HNSCC.

## Methods

### Patients

The eligible patients had a histopathologically proven diagnosis of squamous cell carcinoma of the head and neck region (oral cavity, larynx, oropharynx, hypopharynx); having a recurrence of disease within 6 months of platinum therapy either in primary, recurrent or metastatic setting; having age between 18 and ≤ 70 years with an Eastern Cooperative Oncology Group Performance Status (ECOG PS) of ≤ 2; having normal pre-treatment haematological and biochemical parameters; having financial constraints for targeted and immunotherapy and had radiologically measurable disease. Patients with uncontrolled severe comorbidities, HIV/HBsAg or HCV-related hepatitis and nasopharyngeal carcinoma were excluded.

### Trial design and treatment

This was an investigator-initiated, single-arm, phase II clinical trial conducted in a single centre. Ethical approval from the ethical committee was granted via IECPG-755/30.01.2020 and the CTRI registration was taken (CTRI/2020/02/023378) before the commencement of the study. Patients attending the Head and Neck Clinics of our cancer centre were screened for eligibility. The recruitment period was from January 2020 to December 2021.

The triplet regimen was administered at our centre in the following schedule: Tab. Erlotinib 150 mg once daily; Inj. Methotrexate 40 mg/m^2^ slow i.v. push (D1 & D8) and Inj. 5-Fluorouracil 500 mg/m^2^ i.v. push (D1 & D8). Anti-emetics were administered as premedication which included Inj. Ondansetron 8 mg i.v. push + Inj. Dexamethasone 8 mg i.v. push (D1 & D8) and Tab. Ondansetron 4 mg thrice a day for 3 days, was given as post-medication.

The cycle was repeated every 28 days till progression or unacceptable toxicities. Complete hemogram, kidney and liver function tests were done at each visit (D1 & D8). Patients were followed up clinically at 30 days intervals or earlier in case of toxicity or apparent progression. The response assessment was done at 3 monthly intervals using CECT-face/neck/chest and the response was evaluated using RECIST 1.1. Records of toxicities were maintained and graded using CTCAE v 5 and dose modifications for grade ¾ toxicities were done. After progression, patients received therapy as per the physician’s choice by their performance status.

The quality-of-life analysis was conducted via validated questionnaires of the European Organization for Research and Treatment of Cancer Quality of Life Core Questionnaire (QLQ-C30 v.3) and Quality of Life Head and Neck Module (QLQ-H&N35). The English and Hindi version was used after taking consent from the organisation for use in our clinical trial. Patients were requested to fill the proforma at baseline before initiation of therapy and 3 months after the completion of the therapy. QLQ-C30 included the five functional scales, three symptom scales, a global health status scale and six single domains while QLQ-H&N35 involved eleven single items and seven multi-item scales assessing pain, swallowing (taste and smell), speech, social eating, social contact and sexuality. All single and multi-item domains have a score calculated in a range of 0–100. A higher score for a functional scale, global health status/QoL indicates a high/healthy level of functioning, while it is the opposite for a symptom scale. A higher symptom scale indicates a higher symptom burden, hence a poorer quality of life.

### Study endpoints

The primary endpoint was objective response rate ((overall response rate (ORR) = complete remission (CR) + partial response (PR)) at 3 months. The secondary endpoints were disease control rate (DCR; CR + PR + stable disease (SD)) at 3 months; progression-free survival (PFS) and OS. PFS was calculated from the initiation of treatment until either imaging/clinically confirmed disease progression or death from any cause. OS was calculated from the date of enrolment until death from any cause. In the absence of an event, data were censored on the last day of survival confirmation. Additionally, the patient-reported outcomes (PROs) were assessed at different time points.

### Sample size calculation

The previously reported ORR of second-line chemotherapy for unresectable HNSCC ranged from 0% to 10% [11]. With this background, this trial was performed according to a Simon optimal two-stage design (P0 = 0.10, P1 = 0.30, alpha = 0.05, and beta = 0.200; P0 and P1 are the response proportions of a poor and good drug, respectively [21]. Accepting a type I error of 5% and a power of 80% with 10% dropouts, 30 patients will be planned for enrolment. The number of patients to accrue in stage 1 was 10 (n1 = 10) and 20 in the last stage (stage 2), totally 30 patients were to be tested (n2 = 20). The acceptance points and rejection points in stage 1 were 1 and 5, respectively (a1 = 1, r1 = 5), while in stage 2 it was 6 and 7, respectively, (a2 = 6, r2 = 7).

### Statistical analysis

Data analysis was done using IBM SPSS v.26. Demographic and clinical characteristics were summarised. For the analysis of survival data, Kaplan-Meier curves were constructed, and the log-rank test was used for comparison. Univariate and multivariate survival analyses were performed using Cox proportional hazards model. *p* < 0.05 was considered statistically significant. Repeated measures test was used to check differences in responses with time and association with different variables.

## Results

A total of 45 patients were screened for eligibility to be enrolled in the study. However, ten were excluded due to poor performance status, uncontrolled co-morbidities, unwilling for repeat biopsy and follow-up, and COVID at baseline. Thirty-five patients were recruited in the phase II study. The details of the baseline clinical and laboratory characteristics have been illustrated in Table 1. The median age of the cohort was 45 years (range: 26–65 years), with a male preponderance of 80%. The buccal mucosa (62.9%) was the most involved primary site followed by the tongue. Other sites of involvement were pyriform sinus, maxillary sinus, larynx and retromolar trigone. All 35 patients (100%) received platinum chemotherapy as part of initial therapy either in a curative or palliative setting. Prior radiation was received by 60%. Each cycle was administered every 28 days and continued till progression or unacceptable toxicities. The median number of cycles received was 5 (range: 1–12).

### Efficacy

**Figure 1.**
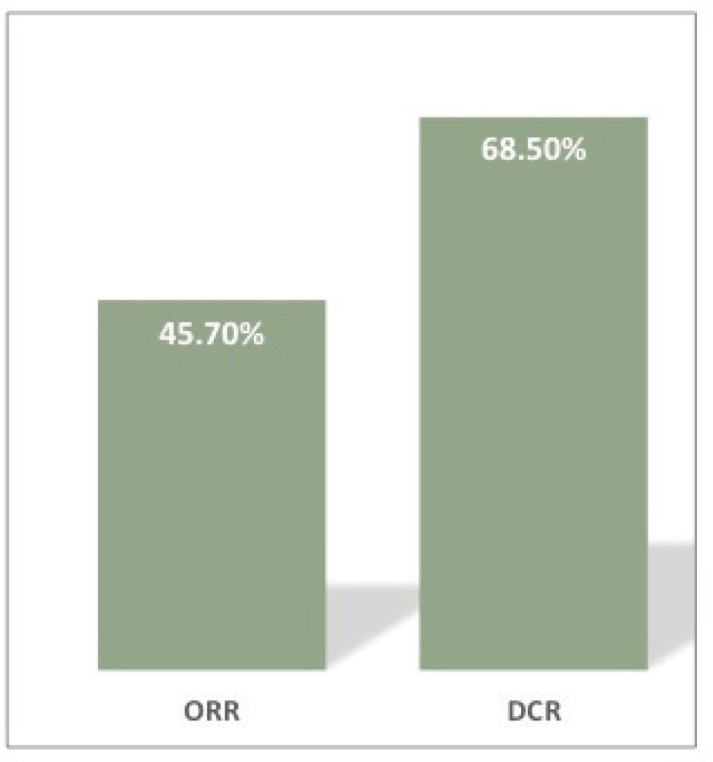
Response rates at 3 months. ORR, Overall response rate – 45.7%; DCR, Disease control rate – 68.5%.

Response assessment via clinical and imaging was done at 3 months. PR seen in 45.7% (16/35), SD in 22.86% (8/35) and progressive disease (PD) in 31.4% (11/35) of the patients. The ORR and DCR at 3 months were 45.7% and 68.5%, respectively (Figure 1).

**Figure 2.**
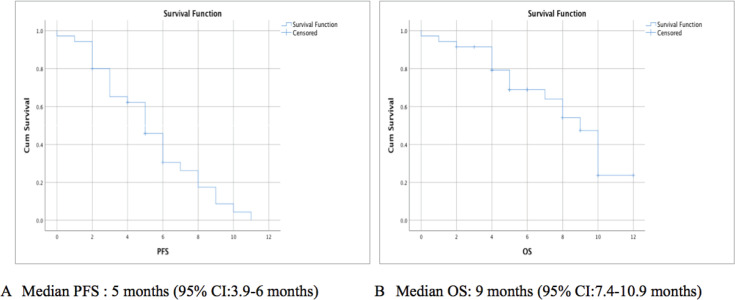
Kaplan-Meir survival curve showing (a): PFS, (b): OS.

On further follow-up, at 6 months PR was seen in 2 (5.8%), SD in 7 (20.5%), PD in 23 (67%), while 2 patients had evaluation pending. The DCR at 6 months was 26.3%. After a median follow-up period of 8 months (95% confidence interval (95% CI): 5–10.9) the median PFS seen was 5 months (95% CI: 3.9–6 months) (Figure 2a). The 3-month and 6-month PFS rate was 86% ± 6% and 45% ± 9%, respectively. The median OS seen was 9 months (95% CI: 7.4–10.9 months) (Figure 2b). The 3-month and 6-month OS rate was 91% ± 5% and 68% ± 8%, respectively.

Further Cox-regression model (Supplementary Table 1) for univariate and multivariate analysis was performed. There was a significant difference seen in PFS between males and females (*p* = 0.046) on univariate analysis; however, it was not statistically significant on multivariate analysis (*p* = 0.730). Other factors like ECOG PS, smoking/tobacco, the primary site of lesion, haemoglobin and albumin were also analysed for association with PFS and OS; however, none was found significant.

### Safety

The various clinical and laboratory treatment-related adverse events have been detailed in Table 2. Skin rash was the most encountered (65%) side effect in our patients. Grade 3/4 toxicity was seen in 14.2% requiring temporary stoppage till it resolved to grade 1 followed by dose reduction. The next frequently observed events were mucositis, fatigue, diarrhoea, decreased appetite, and nausea/vomiting. Grade 3 anaemia was seen in one patient. The dose modifications for the toxicities were made by the standard guidelines (details in Supplementary Tables 2 and 3).

### Compliance and challenges

The compliance with treatment was 88.5% (31/35). Major hurdles were during the COVID-19 lockdown. After a median follow-up of 8 months, 29 patients had progressive disease**,** out of which 16 patients had expired. The subsequent treatment given, post-progression on EMF regimen has been elaborated in Table 3. The most common immediate cause of death was tumour bleed seen in six patients followed by infections seen in four patients. Other causes included brain metastasis and hypercalcaemia, while it was sepsis in four patients.

### Patient-reported outcomes

**Figure 3.**
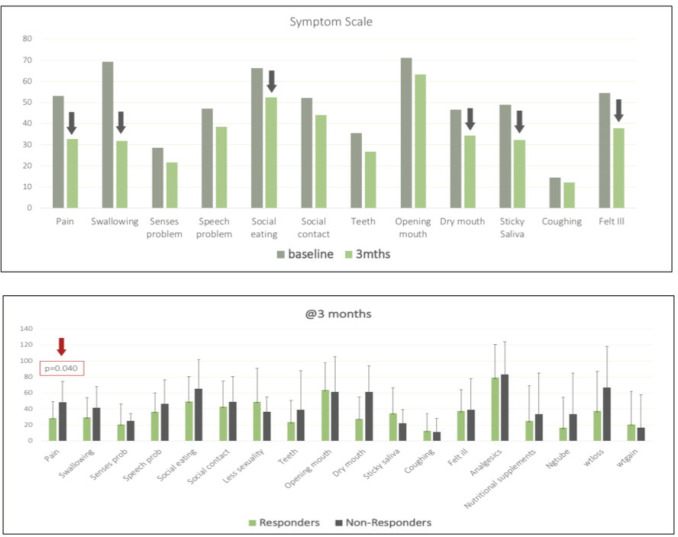
(a): Bar graph showing differences in QLQ C.30 domains at 0 and 3 months, arrows indicate domain with clinically relevant improvement. (b): Bar graph with QLQ H&N35 scores of all domains at 3 months between responders and non-responders, statistically significant difference seen in the domain of pain (*p* = 0.040).

There was a clinically relevant improvement (CRI: difference of ten units from baseline) seen across the following domains of QLQ-C.30 post 3 months of EMF therapy (Supplementary Table 4a): role functioning, emotional functioning, social functioning, fatigue, nausea/vomiting, pain, insomnia, appetite loss, financial difficulties and in global health scale [22, 23]. There was no statistically significant difference between responders and non-responders on the repeated measures test concerning all domains. Similarly, QLQ-H&N35 (Figure 3a) also showed decreased disease burden (lower scores) which were clinically significant in terms of pain, swallowing, social eating, dry mouth, sticky saliva and feeling ill (Supplementary Table 4b). Repeated measures test showed statistically significant improvement in pain between responders and non-responders at 3 months (*p* = 0.040), while the rest of the domain did not yield similar results.

## Discussion

Platinum refractory/resistant disease of the head and neck region has a poor prognosis and limited therapeutic options [9, 13]. Our study showed that the triplet regimen of EMF yielded an ORR and DCR of 45.7% and 68.5%, respectively, at 3 months in patients with HNSCC who had disease progression post-platinum-based chemotherapy. The median PFS seen was 5 months (95% CI: 3.9–6 months) and the median OS seen was 9 months (95% CI: 7.4–10.9 months). The study reported no new safety concerns, was well tolerated and the toxicity profile was similar to that reported in previous studies [9, 24].

Many Indian studies have demonstrated variable responses with different chemotherapeutic agents and oral metronomic therapy in this setting (Supplementary Table 5). Patil *et al* [9] reported 3-month PFS rate of 71.1% and a 6-month OS rate of 61.2% with erlotinib + methotrexate + celecoxib prospectively [9]. Our study with the EMF regimen showed comparable and favourable responses in a similar cohort of platinum-refractory patients with 3-month ORR of 45.7% and 3-month PFS rate of 86% ± 6% and 6-month OS rate of 68% ± 8%. Vivek *et al* showed cetuximab-based chemotherapy to have significant improvement in OS (HR: 0.58; 95% CI: 0.35–0.95, *p* = 0.031) as compared to metronomic chemotherapy in relapsed/metastatic setting [24]. However, our population is laid back to procure the benefits of targeted therapy like cetuximab due to financial constraints. Also, the median OS of 9 months seen with the EMF regimen in the second line in platinum-refractory patients is at par with that seen in the paclitaxel-cetuximab group [8]. EMF regimen showed better response rates and survival rates compared to capecitabine and methotrexate [25]. Intravenous administration would have combatted the obstacles faced due to trismus, difficulty in swallowing and pain which is encountered commonly. Other studies retrospectively evaluated oral metronomic; however, the setting is unclear if it was in upfront metastatic or platinum-refractory disease.

Immunotherapy has also been employed in patients with recurrent HNSCC having early relapses (within 3–6 months) after treatment with platinum-based therapy. CHECKMATE 141 and KEYNOTE 040 demonstrated improved survival with nivolumab and pembrolizumab, respectively, as compared with standard therapy (docetaxel/methotrexate/cetuximab) [13, 26]. The median OS was in the range of 7.5–8.4 months, while the median PFS hovered at 2–2.5 months in the studies mentioned above. We don’t have a randomised control trial comparing EMF to immunotherapy but on the cross-trial comparison, EMF was better with favourable survival outcomes (median PFS – 5 months and median OS – 9 months). Considering the heavy burden of disease, risk of hyper-progression and the exorbitant expenses involved, EMF seems to have succeeded in giving a reasonable, effective, safe therapeutic option.

PROs showed stability in various domains of QOL analysis after treatment with the triplet therapy. There was a clinically relevant improvement in functional, symptom and global health scales at 3 months as assessed by QLQ-C.30 and H&N 35 modules. Responders, as well as non-responders, seem to benefit equally from the triplet therapy. This may be attributed to the placebo effect, and psychological impact of the intravenous therapy, though organically response was not elicited. However, a statistically significant decline in pain (*p* = 0.040) was evident in responders (Figure 3b). Our outcomes are akin to that seen in studies by Vijay *et al*, Kalaichelvi *et al* and Patil *et al* [2] which focused on improvement in QOL in head and neck cancer patients with oral metronomic [9, 24].

There were a few limitations of our study. It was a single-arm study with small sample size, and a comparator arm would add more value. A phase III study comparing EMF with standard chemotherapy regimens in platinum-refractory/resistant HNSCC can be carried out for robust data in the second line. Improved QOL in non-responders highlights the limitation of PROs, owing to the interplay of chemotherapy and supportive care clouding the interpretation.

## Conclusion

The study demonstrated that the triplet regimen of EMF is feasible and safe. It has shown a favourable response in platinum-refractory HNSCC, comparable with available data proving to be an effective regimen. It also showed overall improvement in QOL in all domains, which was pertinent to all patients.

## Conflicts of interest

The authors have no conflicts of interest.

## Clinical trial registration

CTRI/2020/02/023378

## Ethical approval

The study protocol was approved by the Institute Ethics Committee vide letter number: IECPG-755/30.01.2020.

## Financial disclosure

Institute’s resources were utilised. Tab Erlotinib was donated by NATCO Pharma Ltd, India.

## Consent to participate

Informed consent was obtained from all patients.

## Availability of data and material

Data regarding this study will be available from the corresponding author (RP) on reasonable request.

## Authors’ contributions

Study concept/study design/data acquisition: RP/AS/AKB.

Quality control of data and algorithm: AKB/RP

Data analysis and interpretation: RP/AKB

Statistical analysis: AKB/RP

Manuscript preparation: AKB/RP

Manuscript editing/review: RP/AS/AKB/ AB/ SJB/SB/AT/RK/MS

The study abstract was accepted in ASCO 2022 for online publication.

## Figures and Tables

**Supplementary Table 1.** Cox regression analysis.

	PFS			OS		
Variable	Univariate analysis		Multivariate analysis	Univariate analysis		Multivariate analysis (*p* value)
	HR (95% CI)	*p* value		HR (95% CI)	*p* value	
Gender -Male -Female	2.5 (1.0–6.2)	**0.046**	0.561	1.9 (0.68–5.5)	0.215	0.730
ECOG PS -1 ->1	1.05 (0.47–2.32)	0.898	0.39	2.5 (0.96–6.5)	0.060	0.151
Smoking/tobacco -Yes -No	0.59 (0.139–2.57)	0.491	0.168	0.92 (0.12–7.1)	0.939	0.989
Site -Buccal mucosa -Others	1.1 (0.51–2.41)	0.773	0.170	1.44 (0.55–3.77)	0.45	0.562
Haemoglobin <10 g/dL >10 g/dL	1.85 (0.69–4.94)	0.220	0.207	0.949 (0.30–2.94)	0.928	0.779
Albumin <3.5 >3.5	0.20 (0.023–1.8)	0.156	0.210	0.10 (0.09–1.10)	0.060	0.463

**Supplementary Table 2.** Dose interruptions and modifications done in grade 3 rash.

Rash	Erlotinib W/H till grade 1	Dose interruption	Final dose
Patient 1	✓	7 days	Decreased by 50 mg
Patient 2	✓	10 days	Alternate days dosing
Patient 3	✓	7 days	Decreased by 50 mg
Patient 4	✓	5 days	Decreased by 50 mg
Patient 5	✓	12 days	50 mg daily

**Supplementary Table 3.** Dose interruptions and modifications done in other grade 3/4 adverse events.

Parameter	*N*	Days withheld (median)	Dose modifications
Diarrhoea	2	6 (Range: 5–7)	5-FU reduced to 75% dose, erlotinib withheld till symptoms resolved
Mucositis	1	7	Drugs withheld till grade 1
Infection Paronychia Folliculitis	5	7 (Range:7–14)	W/H erlotinib till infection subsided, dose reduction done by 50 mg
Anaemia	1	7	W/H all drugs, dose reduction by 25% 1 PRBC transfusion Hematinics

**Supplementary Table 4. a):** QLQ-C.30 module: Table showing absolute difference between baseline (A0) and at 3 months (A3). Reverse scoring done for symptom scale.

Domain	Baseline (A0) mean ± standard deviation	AT 3 months (A3) mean ± standard deviation	Difference of MEAN (A3–A0)	95% CI		*p* value
				Lower	Upper	
Physical functioning	57.20 ± 23.46	63.871 ± 25.63	6.66667	1.3465	11.98683	0.016
Role functioning	51.61 ± 31.13	63.97 ± 27.58	12.36559	5.1377	19.59349	0.002
Emotional functioning	40.59 ± 28.44	52.15 ± 28.94	11.55914	5.01861	18.09967	0.001
Cognitive functioning	65.59 ± 30.1	74.73 ± 25.77	9.13978	2.28867	15.9909	0.011
Social functioning	46.23 ± 32.11	59.13 ± 31.57	12.90323	5.05684	20.74961	0.002
Fatigue	35.48 ± 26.51	50.17 ± 30.47	14.69534	6.65447	22.73621	0.001
Nausea/vomiting	69.35 ± 25.12	81.18 ±19.5	11.82796	2.33172	21.32419	0.016
Pain	30.10 ± 26.32	53.76 ± 26.42	23.65591	−5.18876	32.12307	<0.05
Dyspnoea	82.79 ± 27.04	86.02 ± 20.68	3.22581	1.61867	8.07028	0.184
Insomnia	48.38 ± 33.15	59.13 ± 28.16	10.75269	0.57819	20.92719	0.039
Appetite loss	43.01 ± 33.54	59.13 ± 28.16	16.12903	3.95497	28.3031	0.011
Constipation	63.44 ± 33.54	70.96 ± 30.68	7.52688	2.8044	7.85816	0.147
Diarrhoea	78.49 ± 32.8	79.56 ± 29.41	1.07527	5.60959	7.76013	0.745
Financial difficulties	32.25 ± 35.9	61.29 ±34.5	29.03226	17.7633	40.30121	<0.05
Global health scale	30.10 ± 22.11	48.38 ± 22.09	18.27957	12.40418	24.15496	<0.05

**b):** QLQ-H&N35 module: Table showing absolute difference between baseline (A0) and at three months (A3).

Domain	Baseline (A0) mean ± standard deviation	At 3 months (A3) mean ± standard deviation	Difference of mean (A3–A0)	95% CI		*p* value
				Lower	Upper	
Pain	53.05 ± 24.70	32.77 ± 22.52	−20.27778	13.55487	27.00068	<0.05
Swallowing	69.25 ± 33.98	31.94 ± 24.96	−37.31481	29.40284	45.22679	<0.05
Senses problem	28.59 ± 28.88	21.66 ± 22.80	−7.22222	2.69198	11.75246	0.003
Speech problem	47.037 ± 30.1	38.51 ± 24.54	−8.51852	2.00992	15.02712	0.012
Social eating	66.38 ± 32.11	52.5 ± 32.39	−13.88889	6.12212	21.65565	0.001
Social contact	52.22 ± 30.12	44 ± 31.63	−8.22222	3.56809	12.87636	0.001
Teeth	35.55 ± 38.08	26.67 ± 32.04	−8.88889	2.40618	15.3716	0.009
Opening mouth	71.11 ± 35.80	63.3 ± 35.39	−7.77778	−0.01474	15.57029	0.05
Dry mouth	46.67 ± 40.68	34.44 ± 30.92	−12.22222	3.28045	21.164	0.009
Sticky saliva	48.88 ± 36.86	32.22 ± 29.66	−16.66667	5.4621	27.87123	0.005
Coughing	14.44 ± 24.26	12.22 ± 20.49	−2.22222	−0.93565	5.38009	0.161
Felt Ill	54.44 ± 33.31	37.77 ± 28.67	−16.66667	5.94949	27.38384	0.003

**Supplementary Table 5.** Data available for metronomic therapy from different trials in relapsed/recurrent HNSCC in India.

Trials	Regimen	Setting	RR	PFS
Patil *et al* [1] *N* = 110	Celecoxib + MTX versus CDDP	72% – 2^nd^ line 28% – upfront	12.3% versus 1.9%	101 versus 66 days
Mateen *et al* [25] *N* = 72	MTX + capecitabine	2^nd^ line, interval of >6 months of chemo	PR – 29% SD – 54%	0.67 years
Geeta *et al* [24] *N* = 15	Erlotinib + celecoxib + MTX	Prospective unclear	CR – 13.3% PR – 46% SD – 13.3%	148 days
Kalaichelvi *et al* [24] *N* = 30	Celecoxib + MTX	Retrospective unclear	70% clinical benefit rate @ 4 months	
Pai *et al* [27] *N* = 32	Celecoxib + MTX	Retrospective, upfront Waiting period > 3 weeks	2 year DFS – 94.6% versus 75.4%	
Shobana *et al* [24] *N* = 13	Erlotinib + MTX + celecoxib	Recurrent/metastatic unclear	-	153 days
Patil *et al* [9] *N* = 76	Erlotinib + MTX + celecoxib	2nd	RR – 42.9%	3 month PFS – 71% 6 month OS – 61%
Our study *N* = 35	Erlotinib + MTX + 5-FU	2^nd^/3rd	ORR at 3 months: 45.7% DCR at 3 months: 68.5%	Median PFS: 5 months Median OS: 9 months

**Table 1.** Baseline clinical and laboratory characteristics.

Variables	*N* (%)
Age, median & range (years)	45 (26–65 years)
Sex – Male – Female	28 (80%) 7 (20%)
ECOG PS – 1 – 2	23 (65.7%) 12 (34.3%)
Smoking/tobacco – Yes – No	33 (94.3%) 2 (5.7%)
Primary site – Buccal mucosa – Lateral border of tongue – Base of tongue – Others	22 (62.9%) 3 (8.6%) 5 (14.3%) 5 (14.3%)
Extent – Locoregional – Local & distant metastasis (lungs)	30 (85.7%) 5 (14.2%)
Lines of previous therapy – 1 – >1	34 (97.1%) 1 (0.02%)
Previous exposure to: – Platinum – Paclitaxel – Cetuximab – Immunotherapy	35 (100%) 13 (37%) 1 (0.02%) 1 (0.02%)
Previous radiation – Yes – No	21 (60%) 14 (40%)
**Laboratory characteristics**	
**Variable**	**Median (range)**
Total leucocyte count (/mm^3^) Median (range)	7,590 (3,460–13,180)
Platelets (× 10^5^/dL) Median (range)	3.22 (1.15–9.07)
Creatinine (mg/dL)	0.70 (0.3–1)
AST (U/L) ALT (U/L)	23 (12–45) 20 (7–63)
Albumin (gm/dL)	4.2 (3.2–5.3)
ALP (IU/L)	101 (61–420)

ECOG PS, Eastern Cooperative Oncology Group Performance status; AST, Aspartate amino transferase; ALT, Alanine amino transferase; ALP, Alkaline phosphatase

**Table 2.** Treatment related clinical and laboratory adverse event of any grade.

Parameter	Any grade: *N* (%)	Grade 3/4
Clinical		
Rash	23 (65%)	5 (14.2%)
Diarrhoea	6 (17%)	2 (5.7%)
Fatigue	9 (25.7%)	1 (2.8%)
Mucositis	14 (40%)	1 (2.8%)
Nausea/vomiting	3 (8%)	0%
Decreased appetite	5 (14.2%)	0%
Infections		
Paronychia	1 (2.8%)	
Pustular lesions/folliculitis	4 (11.8%)	
URI/LRTI	3 (8%)	
Laboratory parameter	Any grade: *N* (%)	Grade 3
Anaemia	4 (11.4%)	1 (2.8%)
Neutropenia	3 (8%)	-
Thrombocytopenia	4 (11.4%	-
Transaminitis	7 (20%)	-
Deranged creatinine	3 (8%)	-
Increased bilirubin	2 (5%)	

**Table 3.** Subsequent treatment after progression on EMF regimen.

Modalities	*N* = 29
Hemostatic/palliative RT	3
Chemotherapy	3
Immunotherapy	1
Alternative medications	2
Best supportive care	20
